# Gynæcology

**Published:** 1878-08

**Authors:** 


### GYNÆCOLOGY.

THE INFLUENCE OF THE UTERUS IN EYE DISEASES. Swanzy. (*Obstetric Journal*, May, 1878.)

The author read a paper with this title before the Obstetric Society, of Dublin. The first disease which he mentioned, as probably having its cause in the uterus, was iritis, occurring in girls from the eleventh to the seventeenth year. He had seen but few cases, but in these few he could trace no other cause; iritis is extremely rare at this age, unless depending on syphilitic or corneal diseases, and he had never seen similar cases in the male. In all these cases there was little or no pain, vascular injection or photophobia. The anterior chamber usually remained clear, and there was no deposit upon the cornea, but there was a tendency to formation of posterior synechia. Indications that the ciliary body and the choroid were implicated were also present. The affection was slow, difficult to cure, and apt to recur. The gravest uterine symptom he had discovered was insufficient menstrual flow.

Retinitis and optic neuritis may depend on disturbances of menstruation. Mandelstamm, Von Graefe and Mooren had seen such cases, and he was of the opinion that retroflexions and ovarian tumors might play a role in their ætiology.

Retinal appoplexies are sometimes the consequence of cessation or suppression of the menses. Liebreich had described and figured such cases, and the author had observed them.

Atrophy of the optic nerve had been repeatedly noted by Pagenstecher as occurring in women who suffered from severe menstrual disturbances.

That form of asthenopia called kopiopia hysterica must now be considered as simply a symptom of uterine disease. Förster had described it at length and Freund had invariably found *post mortem* that pathological uterine lesions accompanied it. Patients complain of inability to use their eyes for near work owing to darting pains, and these pains are augmented by any depressing circumstances; they are troubled with photophobia, especially of artificial light at night. No organic disease of the eye exists, but hypermetropia or insufficiency of internal recti may mislead the surgeon. The eyes are never injected, there is no swelling of the lids, no epiphora; such patients have good and bad days, and before and during menstruation such symptoms are more severe. According to Freund the uterine lesion was a chronic inflammatory process attacking the parametrium, sometimes producing atrophy of that tissue with various displacements. The course is a long chronic one; painful regions are often found in the pelvis, and there is more or less venous congestion of the vagina, with chronic metritis, catarrh and ulceration. The best treatment is attention to the general health.

Besides the five affections metioned above as apparently depending on uterine derangement, it was probable that others might be mentioned, but the author did not wish to fall into the error of regarding every eye disease as dependent upon such derangement merely because it happened consentaneously.

SALICYLIC ACID IN LEUCORRHŒA. (*L' Union Méd. du Canada*, June, 1878.)

In the *Revue Medico-Chirurgicale* of Buenos-Ayres, Dr. Y.

P. recommends the use of salicylic acid in this affection. The author says he has employed injections of the acid with completely satisfactory results for chlorotic women of lymphatic constitution, in vaginitis, in discharges from the uterine mucous membrane in which the irritant fluid produces erosion of the neck and vulvar inflammation. His formula is the following: salicylic acid 6 Gms., glycerine 100 Gms. Dissolve over water bath and add a liter of water (for six injections, one a day).

In case of uterine leucorrhœas an irrigating apparatus should be employed so as to project a small quantity into the uterus. The solution of the acid should be complete, otherwise the undissolved crystals might be irritating. The glycerine facilitates solution.

In a case that had resisted other treatment, a tampon of cotton, soaked in a two per cent. solution of salicylate of zinc was left in the vagina 24 hours, after which a one per cent. solution of the same salt, containing a little morphine, was used three times a day for a week, by injection, and followed by complete cure.

